# Synchronization of the ovulation and copulation timings increased the number of *in vivo* fertilized oocytes in superovulated female mice

**DOI:** 10.1371/journal.pone.0281330

**Published:** 2023-02-06

**Authors:** Satohiro Nakao, Kotono Ito, Chihiro Sugahara, Hitomi Watanabe, Gen Kondoh, Naomi Nakagata, Toru Takeo

**Affiliations:** 1 Division of Reproductive Engineering, Center for Animal Resources and Development, Kumamoto University, Kumamoto, Japan; 2 Laboratory of Integrative Biological Science, Institute for Life and Medical Sciences, Kyoto University, Kyoto, Japan; 3 Division of Reproductive Biotechnology and Innovation, Center for Animal Resources and Development, Kumamoto University, Kumamoto, Japan; University of Florida, UNITED STATES

## Abstract

The number of sperm that reaches the oocytes in mammalian species is limited. In mice, 8–10 oocytes are ovulated, a similar number of sperm reaches the oocytes, and nearly all oocytes are fertilized via natural mating. Meanwhile, our improved superovulation technique (ultrasuperovulation: administration of inhibin antiserum and equine chorionic gonadotropin [IASe]) produced 100 oocytes from a single female C57BL/6 mouse but resulted in only approximately 20 fertilized oocytes via mating. We hypothesized that sperm shortage in the ampulla might cause this low fertilization rate. Mice were mated in the proestrus stage or after hormone injection, but ovulation timing was not considered. In clinical application, the rhythm method supports fertilization by testing the ovulation period and synchronizing the ovulation and copulation timings. Therefore, this study examined the effects of ovulation and copulation timings on *in vivo* fertilization in female mice with IASe. Synchronization of the ovulation and copulation timings increased fertilization efficiency in female mice with ultrasuperovulation. The number of embryos obtained post ovulation was three times higher than that obtained pre ovulation. This study suggests that synchronized ovulation and copulation timings improve the efficiency of *in vivo* fertilization in IASe-treated female mice. This technique can be used to produce genetically modified mice and develop technologies for infertility treatment.

## Introduction

In mammals, the ejaculated sperm in the vagina travels through the uterus to the oviduct and reaches the oocyte. During this process, a limited number of sperm can enter the ampullary region of the oviduct before fertilization [1, 2]. This process has been observed in mice by visualizing sperm migration [3–6].

Mature female mice typically ovulate 8–10 oocytes via spontaneous ovulation, and nearly all oocytes are fertilized via mating [7]. Meanwhile, artificial induction of superovulation using equine chorionic gonadotropin (eCG) and human chorionic gonadotropin (hCG) increases the number of ovulated oocytes (approximately 20 oocytes per female). Subsequent mating can produce nearly 20 fertilized oocytes [8–12].

Previously, we developed an improved superovulation method (ultrasuperovulation) using inhibin antiserum and eCG (IASe), which produced approximately 100 oocytes from a single C57BL/6 (B6) female mouse [13]. However, ovulation induced by IASe only resulted in approximately 20 fertilized oocytes, despite the large number of ovulated oocytes in the oviductal ampulla [14]. This result suggests that a limited number of sperm reach the ampulla in natural mating. Assisted reproductive technologies (ARTs) can improve fertilization rates, but whether sperm limits can be exceeded with ARTs remains unknown.

In clinical application, the rhythm method is widely used for fertility treatment to test the ovulation period, and copulation during ovulation increases the pregnancy rate [15]. Generally, mouse mating is performed by simply cohabiting female mice in the proestrus stage with mature male mice overnight [16] and does not consider the ovulation timing. Ovulated oocytes in mice are produced 13–16 h after hCG administration via the superovulation technique [17], and the timing of ovulation can be accurately predicted.

In this study, we first examined the effect of ovulation and copulation timings on the fertilization of B6 female mice. We examined the fertilization rate *in vivo* and divided it into three copulation timings: pre ovulation, during ovulation, and post ovulation. Furthermore, the oocytes derived from IASe are widely used for fertilized oocyte production via *in vitro* fertilization (IVF) and live pup production via embryo transfer [18]. In this study, *in vivo*-derived fertilized oocytes were produced via IASe-induced ovulation; hence, we compared the developmental ability of fertilized oocytes derived *in vivo* and *in vitro*.

## Materials and methods

### Animals

Male (12–18 weeks old) and female (4 weeks old) C57BL/6J mice (CLEA Japan, Tokyo, Japan) were used for *in vitro* fertilization and copulation. Female Jcl:ICR mice (>9 weeks old; CLEA Japan) were used as embryo transfer recipients. The mice were housed under a 12-h dark/light cycle (light period from 7:00 to 19:00) at 22 ± 2°C with free access to food and water. The Animal Care and Use Committee of Kumamoto University approved the study protocols. All experiments were performed in accordance with relevant guidelines and regulations.

### Media

IAS was produced from goats immunized with an inhibin-specific peptide coupled with keyhole limpet hemocyanin [19]. Equine chorionic gonadotropin (eCG) and human chorionic gonadotropin (hCG) were purchased from ASKA Animal Health Co. Ltd., Japan. A mixture of IAS and eCG (IASe) was used for superovulation. IASe is available from COSMO BIO Ltd. (CARD HyperOva, catalog number: KYD-010-EX). Bovine serum albumin-free Toyoda-Yokoyama-Hosi medium (TYH) containing 0.75 mM of methyl-β-cyclodextrin and 1.0 mg/mL of polyvinyl alcohol (cTYH) was used to preincubate the sperm (S1 Table) [20–22]. Modified human tubal fluid (mHTF) was used as a medium for IVF [23, 24], and potassium simplex medium (KSOM) was used for embryo culture and transfer (S1 Table) [25].

### Ultrasuperovulation

Four-week-old female mice were intraperitoneally injected with IASe (0.1 mL of IAS and 3.75 IU of eCG). Approximately 48 h after the IASe injection, the mice were intraperitoneally injected with 7.5 IU of hCG. Both injections were administered at 18:00.

### Assessment of ovulation time

Female mice with induced ultrasuperovulation were sacrificed via cervical dislocation. Their oviducts and ovaries were collected into liquid paraffin in a petri dish and were observed under a stereo microscope. A dissecting needle was used to tear open the ampullae of the oviducts, and cumulus-oocyte complexes (COCs) were collected into a 200-μL mHTF drop. Approximately 20 μL of 1% (10 mg/mL) hyaluronidase was added to the 200-μL mHTF drop containing COCs, and the dish was kept at 37°C for 1 min in a 5% CO_2_ incubator. Cumulus cells were removed by washing the oocytes in the 100-μL mHTF drop thrice. Morphologically normal oocytes were counted.

### Copulation

Male mice were caged individually before injecting female mice with IASe. Male and female mice were co-housed for the following periods: pre ovulation (0–10 h after hCG injection), during ovulation (10–15 h after hCG injection), and post ovulation (15–19 h after hCG injection). After co-housing, the vaginal plug was observed to confirm whether the male and female mice had copulated.

### Embryo flushing

Female mice were sacrificed via cervical dislocation 43–45 h after hCG injection. The oviducts were rinsed in KSOM and collected into small KSOM drops. A flushing needle and a 1-mL syringe were used to flush the embryos from the oviducts. Two-cell embryos were washed twice in 80-μL KSOM drops. The number of morphologically normal two-cell embryos was counted, and the fertilization rate was calculated as follows: fertilization rate (%) = the number of two-cell embryos / the total number of oocytes and two-cell embryos × 100.

### IVF

Female mice were sacrificed via cervical dislocation 15 h after hCG injection. The oviducts were collected into liquid paraffin in a fertilization dish, and the COCs were transferred into a 200-μL mHTF drop. Male mice were sacrificed via cervical dislocation, and the cauda epididymides were collected into liquid paraffin in a preincubation dish. The sperm was collected into a 100-μL cTYH drop using a dissecting needle. The sperm was preincubated at 37°C, 5% CO_2_ for 60 min. After preincubation, a 3-μL sperm suspension was introduced into the fertilization drop containing COCs and incubated. The oocytes were washed in the 80-μL mHTF drop thrice 3 h after insemination. Parthenogenic or polyspermic oocytes were removed 6 h after insemination. The number of two-cell embryos was counted 24 h after insemination, and the fertilization rate was calculated as follows: fertilization rate (%) = the number of two-cell embryos / the number of inseminated oocytes × 100.

### Embryo culture

The two-cell embryos produced by IVF or copulation were washed in 100-μL KSOM drops twice and incubated at 37°C, 5% CO_2_. The numbers of four-cell embryos, morulae, and blastocysts were counted at 48, 72, and 96 h after insemination, respectively. The developmental rate was calculated as follows: developmental rate (%) = the number of embryos in each stage / the number of two-cell embryos.

### Embryo transfer

The two-cell embryos produced via IVF or copulation were transferred into both oviducts (7–10 embryos/oviduct) of pseudopregnant ICR female mice on the day of vaginal plug discovery. At 19 days after the embryo transfer, the number of live pups was counted, and the birth rate was calculated as follows: birth rate (%) = the number of live pups / the number of transferred embryos.

### Statistical analysis

Statistical analysis was performed using Prism version 8.0 (GraphPad Software). The results are expressed as the mean ± standard deviation (SD). The means were compared by analyzing the variance of arcsine transformation of the percentage data. The Tukey–Kramer test was performed on the means, and p-values of <0.05 indicated statistical significance.

## Results and discussion

### Results

#### During ovulatory or postovulatory copulation improved the fertilization rate *in vivo*

We confirmed the timing of ovulation after hCG injection to synchronize the ovulation and copulation timings in IASe-treated female mice. Ovulation started 10 h after hCG injection (S1A–S1F Figs). The number of ovulated oocytes rapidly increased until 12 h and then gradually increased from 12 h to 16 h (S1G Fig).

**Fig 1:**
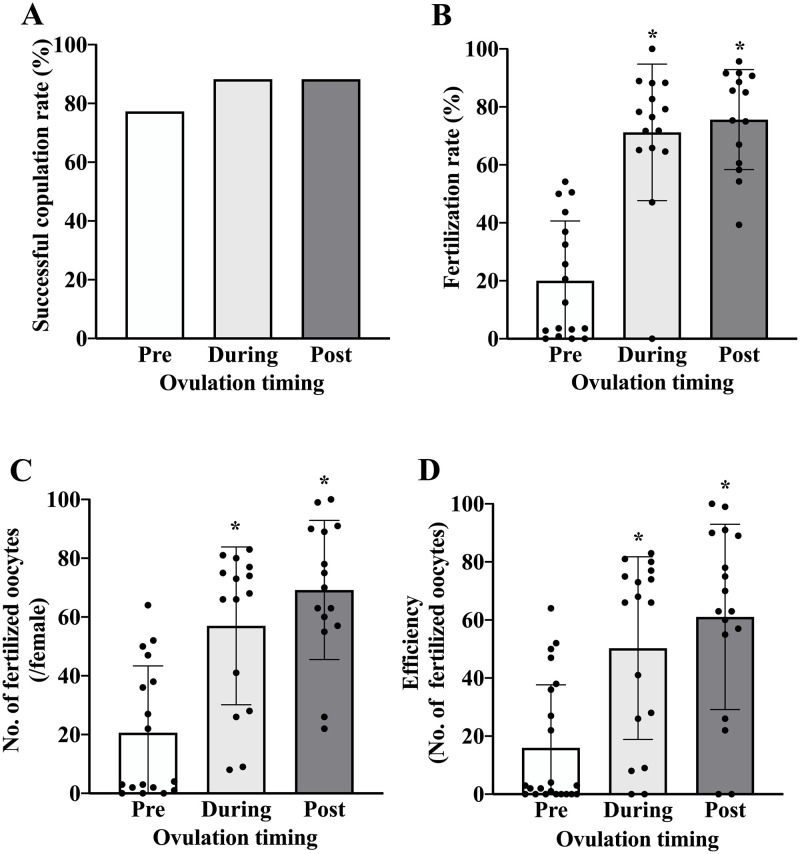
Effect of synchronizing the timings of ovulation and copulation on the fertilization rate *in vivo*. Female mice (4 weeks old) treated with IASe were divided into three groups: pre ovulation (0–10 h after hCG injection), during ovulation (10–15 h after hCG injection), and post ovulation (15–19 h after hCG injection). These female mice were then copulated with male mice in each period. (A) The successful copulation rate was calculated using the following equation: Successful copulation rate (%) = total number of plugged female mice / total number of female mice used for copulation × 100. (B) The fertilization rate was calculated using the following equation: fertilization rate (%) = total number of two-cell embryos / total number of collected oocytes and two-cell embryos × 100. (C) The average number of fertilized oocytes obtained from each female mouse with a plug. (D) The efficiency was the average number of fertilized oocytes obtained from each female mouse used for copulation. Values are presented as the mean ± SD (n = 15–21). *p < 0.05 compared with pre ovulation.

The ovulation timing was divided into three groups: pre ovulation (0–10 h after hCG injection), during ovulation (10–15 h after hCG injection), and post ovulation (15–19 h after hCG injection). Female mice were co-housed with male mice pre ovulation, during ovulation, and post ovulation to examine the effect of copulation timing on the fertilization rate. The successful copulation rate was high in all periods (Fig 1A). The fertilization rates were improved by synchronizing the copulation timing (during ovulation and post ovulation) (Fig 1B). In addition, the number of two-cell embryos obtained from a single female mouse with a plug significantly increased (Fig 1C). The efficiency, i.e., the average number of fertilized oocytes obtained from a single female mouse used for copulation, also significantly increased (Fig 1D). The average number of ovulated oocytes was not significantly different among the pre ovulation, during ovulation, and post ovulation periods (93.3 ± 31.8, 75.4 ± 37.3, and 90.8 ± 23.3, respectively).

#### Two-cell embryos obtained from postovulatory copulation normally developed into blastocysts and live pups

We performed embryo culture *in vitro* and embryo transfer to examine the developmental ability of two-cell embryos produced via IVF or copulation (post ovulation). Two-cell embryos derived via IVF and copulation normally developed into blastocysts in *in vitro* culture (Table 1). Furthermore, two-cell embryos derived via IVF and copulation normally developed into live pups by embryo transfer. The birth rate was slightly higher than that of IVF (Table 2).

**Table 1:** *In vitro* developmental ability of embryos obtained via postovulatory copulation.

	No. of two-cell embryos	No. of four-cell embryos (%)	No. of morulae (%)	No. of blastocysts (%)
**IVF**	222	212 (97.7 ± 3.3)	207 (95.4 ± 5.3)	187 (85.8 ± 4.2)
**Copulation**	308	298 (96.9 ± 2.9)	283 (92.9 ± 4.4)	254 (83.2 ± 11.9)

Values are presented as the mean ± SD (n = 5 or 6). There was no significant difference.

**Table 2:** *In vivo* developmental ability of embryos obtained via postovulatory copulation.

	No. of transferred two-cell embryos	No. of recipients	No. of live pups (%)
**IVF**	235	13	83 (35.3 ± 18.8)
**Copulation**	365	21	180 (49.3 ± 15.0*)

Values are presented as the mean ± SD (n = 13 or 21).

*p < 0.05 compared with IVF

### Discussion

This study found that copulation during ovulation and post ovulation improved the *in vivo* fertilization rate of B6 female mice with ultrasuperovulation. Synchronization of the ovulation and copulation timings increased the number of fertilized oocytes (pre vs. during vs. post: 20.8 ± 22.6 vs. 54.5 ± 27.0 vs. 70.8 ± 24.2, respectively), and the efficiency of fertilized oocyte collection, which was the number of fertilized eggs per all female mice used for copulation, was improved (pre vs. during vs. post: 16.0 ± 21.7 vs. 50.3 ± 31.5 vs. 61.1 ± 31.9, respectively). The developmental ability of fertilized oocytes derived *in vivo* (post ovulation) was higher than that of fertilized oocytes derived *in vitro* (IVF) (*in vivo* vs. *in vitro*: 35.3 ± 18.8% vs. 49.3 ± 15.0%, respectively).

One possible reason for the low *in vivo* fertilization rate when using IASe is the insufficient number of sperm in the oviduct. After mating, millions of sperm are accumulated in the uterus, but only a small number of sperm can pass through the uterotubal junction [3, 4]. Muro *et al*. reported that the mouse sperm reached the uterus and distributed in the uterotubal junction 15 min after coitus, and the number of sperm reaching the ampulla was similar to the number of spontaneously ovulated oocytes (2–6 sperm/oviduct), although accurately determining the number of sperm in the ampulla is difficult [7]. In this study, more number of fertilized oocytes were obtained from female mice with IASe upon synchronizing the ovulation and copulation timings. This result suggests that a sufficient number of sperm reaches the ampulla to fertilize the oocytes. Since mating after ovulation is unnatural, sperm may not undergo the process that occurs in natural mating (sperm remain in the fallopian tube stenosis until ovulation). In other words, once the sperm enter the female’s genital tract, these travel directly up to the oviduct, resulting in a higher number of sperm arriving to the ampulla. However, further experiments are required to observe the number of sperm reaching the ampulla.

Another possible reason is that the number of sperm was sufficient, but the fertilization potential of the sperm was not maintained at the time of fertilization. Smith *et al*. reported that golden hamster sperm introduced by copulation and artificial insemination before ovulation did not reach the ampulla and remained in the lower segments of the isthmus until ovulation began [1]. We hypothesized that the fertilizing ability of sperm might be lost during this waiting period. Several reports have shown that the fertilizing lifespan of mouse sperm in the female reproductive tract is 6–12 h, and the fertilizing ability apparently decreases after 4 h [26, 27]. Synchronizing the ovulation and copulation timings might shorten the sperm waiting time, resulting in a larger number of living sperm. However, sperm migration from the uterus to the ampulla should be observed in future studies.

In this study, synchronization of the ovulation and copulation timings was effective to improve the fertilization rate *in vivo*. The timing of sperm injection after hCG administration in artificial insemination affected the fertilization rate. Sato *et al*. reported that sperm injection 7 h after hCG administration increased the fertilization rate in the intrabursal transfer of mouse sperm [28]. The increased fertilization rate by synchronizing the ovulation and copulation timings or sperm injection may be affected by the optimal environment for fertilization in the oviduct. The ovulation timing affected the speed of fluid flow in the oviduct. Hino *et al*. reported that there was a rapid fluid flow in the oviducts toward the ampulla at the ovulation timing (estrus stage), and sperm ride this stream through the oviduct [29]. These reports support the importance of ovulation and copulation timing or sperm introduction in *in vivo* fertilization. Therefore, synchronizing the ovulation and copulation timings may support the sperm to enter the oviduct and achieve fertilization by riding the oviductal fluid flow. However, further studies are required to confirm this phenomenon.

In our experiment, the developmental ability of embryos derived via copulation was slightly higher than that of embryos derived via IVF (copulation vs. IVF: 49.3 ± 15.0% vs. 35.3 ± 18.8%). Mice embryos cultured *in vitro* have a lower developmental rate in live pups than those cultured *in vivo*, and differences in imprinted genes and differentiation markers were observed [30]. Thompson *et al*. suggested differences in metabolism, morphology, and ultrastructure at the cellular and intracellular levels between bovine embryos derived *in vitro* and *in vivo* [31]. Enright *et al*. reported that fertilized oocytes derived *in vitro* and *in vivo* had an equal developmental ability into blastocysts, whereas embryos freezing tolerance was different in bovine [32]. Moreover, Giritharan *et al*. reported that IVF and embryo culture affect the trophectoderm and inner cell mass transcriptomes [33]. Additional experiments on these points are required to clarify the details of the difference in the developmental ability of embryos produced *in vitro* and *in vivo* using IASe.

There are some reports on delayed mating in mice. Ishikawa *et al*. reported that preimplantation embryos derived from delayed mating (6 h after ovulation, when the estimated ovulation time is midnight) progressed more rapidly than their normally mated counterparts (0 h after ovulation) in 9–11 month old ICR female mice [34]. Sakai and Endo reported that delayed mating (12:00–14:00) decreased the successful copulation rate and no embryos could be obtained in spontaneously ovulated ICR female mice [35]. Marston and Chang also reported on the fertilization lifespan of oocytes and revealed that fertilization rates decreased in female mice that were inseminated 21 h after hCG injection [36]. These results suggest that delayed mating causes a reduction in fertilization rates. Marston reported that the fertilizing lifespan of oocytes was 15 h after ovulation [36]. Iwamoto also reported that the *in vitro* fertilizing lifespan of oocytes was 10–12 h [37]. In this study, we allowed postovulatory copulation at 9:00–13:00, but copulation at later times was not considered. Further experiments on copulation after 13:00 would clarify the possible time period of copulation without adverse effects.

In this study, juvenile B6 female mice (4 weeks old) were used because they had the highest number of ovulated oocytes. We also performed the same experiment on mature B6 female mice (8–14 weeks old), which are used in natural breeding. The successful copulation rate was high in all periods (S2A Fig), and the fertilization rates were improved by synchronizing the copulation timing (during ovulation and post ovulation) (S2B Fig). The number of two-cell embryos obtained from a single female mouse with a plug and the efficiency were also significantly increased (S2C and S2D Figs). The fact that the fertilization efficiency was improved by synchronizing the timing, regardless of using juvenile or mature mice, suggests that this copulation method is a highly versatile technique for the production of *in vivo* fertilized oocytes.

The technique of producing fertilized oocytes via mating will be useful to efficiently produce genetically modified mice. Previously, we reported that IASe-derived fertilized oocytes produced via IVF were applied to genome editing to produce genetically modified mice using microinjection and electroporation [38–40]. Previously, we could not recommend the IASe to produce *in vivo* fertilized oocytes due to the low yield of fertilization by mating. Now, we found that synchronizing the ovulation and copulation timings overcame the yield of fertilization *in vivo*. Therefore, IASe-derived *in vitro* and *in vivo* fertilized oocytes can be selected for experimental studies. Improving the yield of fertilized oocytes will contribute to the 3R principles (replacement, reduction, and refinement) by reducing the number of oocyte donors to obtain the required oocyte number (reduction) [41].

Our study showed that synchronization of ovulation and copulation timings on an hourly basis led to a high fertilization rate. Meanwhile, the rhythm method predicts ovulation on a day-by-day basis using luteinizing hormone blood tests and ultrasound scans [42, 43]. Hourly prediction of ovulation may improve the success rate of fertilization in clinical settings. However, further investigation is needed to precisely predict the ovulation timing and the effect of synchronizing the ovulation and copulation timing in other mammalian species.

## Conclusions

In summary, this study suggests that the synchronization of ovulation and copulation timings improves the efficiency of *in vivo* fertilization in IASe-treated B6 female mice. This finding demonstrates the importance of synchronizing the ovulation and copulation timings for successful fertilization. This technique will apply to genetically modified mice production and technology development for infertility treatment.

## Supporting information

S1 Fig Time of ovulation and the number of ovulated oocytes using ultrasuperovulation. The oviducts, ampulla, and ovary were collected from female mice, and the ovulated oocytes were counted several times after hCG administration. (A–F) Collected ampulla (9 h: A, 10 h: B, 11 h: C) and ovary (9 h: D, 10 h: E, 11 h: F) were observed under a microscope. (G) The number of morphologically normal oocytes was counted. Values are given as the mean ± SD (n = 4).
(PDF)

S2 Fig Effect of synchronizing the timing of ovulation and copulation on fertilization rate *in vivo*. Female mice (8–14 weeks old) treated with IASe were divided into three groups: pre ovulation (0–10 h after hCG injection), during ovulation (10–15 h after hCG injection), and post ovulation (15–19 h after hCG injection). These female mice were then allowed to copulate with male mice in each period. (A) The successful copulation rate was calculated using the following equation: successful copulation rate (%) = total number of plugged female mice / total number of female mice used for copulation × 100. (B) The fertilization rate was calculated using the following equation: fertilization rate (%) = total number of two-cell embryos / total number of collected morphologically normal oocytes × 100. (C) The average number of fertilized oocytes obtained from each female mouse with a plug. (D) The efficiency was the average number of obtained fertilized oocytes from each female mouse used for copulation. Values are presented as the mean ± SD (n = 15–17). *p < 0.05 compared with pre ovulation.
(PDF)

S1 Table Composition of the media. (DOCX)
