# Supplementary figures and images for: Synchronization of the ovulation and copulation timings increased the number of in vivo fertilized oocytes in superovulated female mice
Source: PLoS One. 2023 Feb 6;18(2):e0281330. doi: 10.1371/journal.pone.0281330 (PMC9901804; doi:10.1371/journal.pone.0281330)

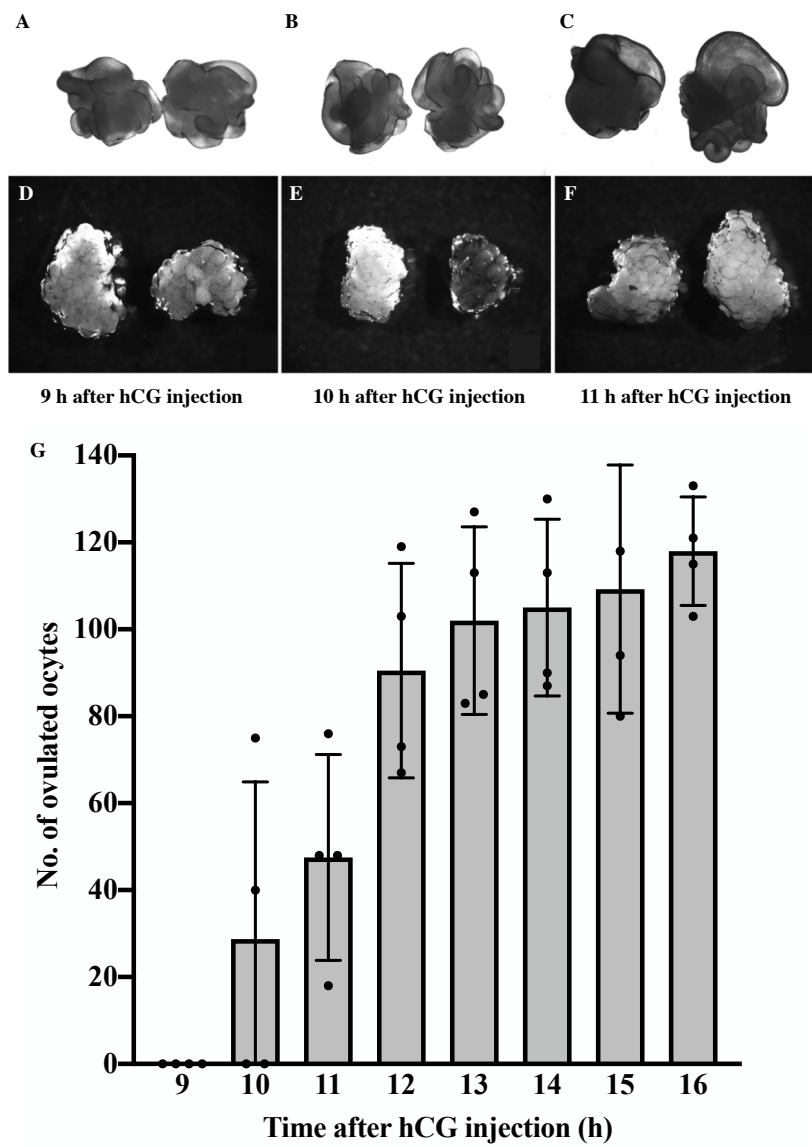

S1 Fig Nakao et al.

Supplement: S1 Fig — The oviducts, ampulla, and ovary were collected from female mice, and the ovulated oocytes were counted several times after hCG administration. (A–F) Collected ampulla (9 h: A, 10 h: B, 11 h: C) and ovary (9 h: D, 10 h: E, 11 h: F) were observed under a microscope. (G) The number of morphologically normal oocytes was counted. Values are given as the mean ± SD (n = 4). (PDF) [file pone.0281330.s001.pdf]

**A**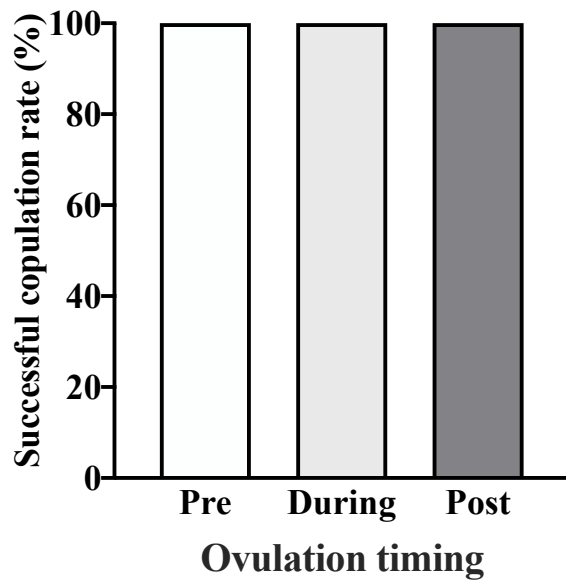**B**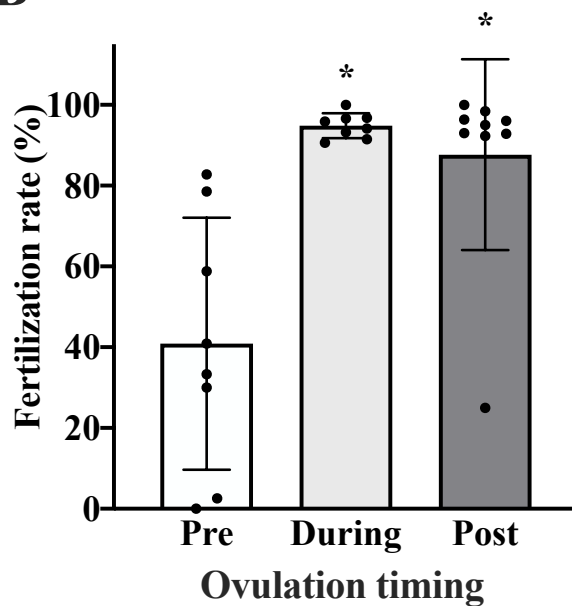**C**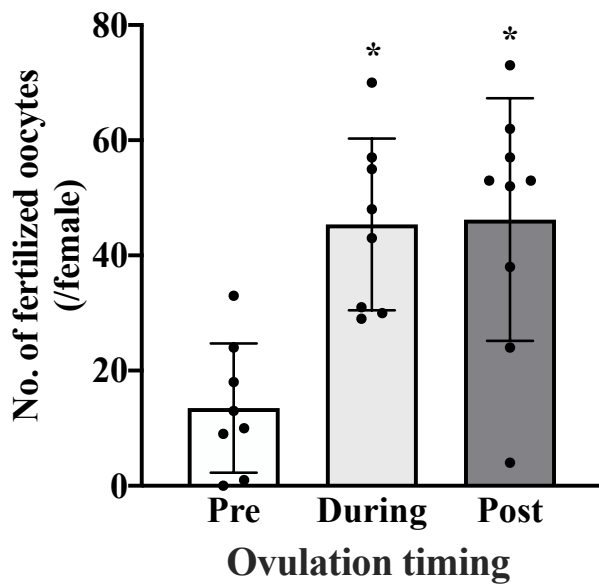**D**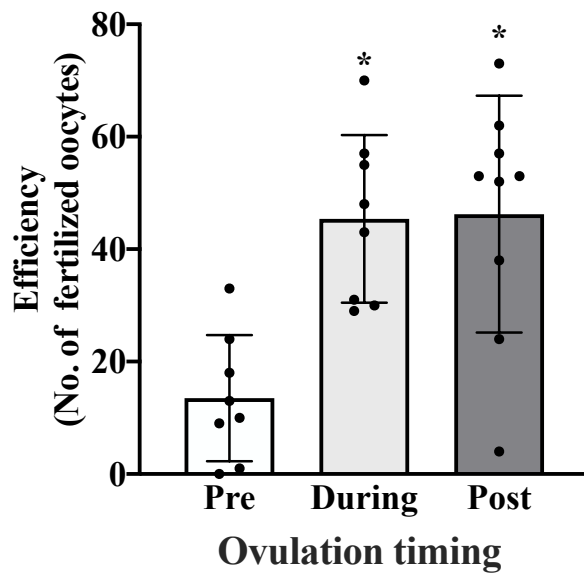

Supplement: S2 Fig — Female mice (8–14 weeks old) treated with IASe were divided into three groups: pre ovulation (0–10 h after hCG injection), during ovulation (10–15 h after hCG injection), and post ovulation (15–19 h after hCG injection). These female mice were then allowed to copulate with male mice in each period. (A) The successful copulation rate was calculated using the following equation: successful copulation rate (%) = total number of plugged female mice / total number of female mice used for copulation × 100. (B) The fertilization rate was calculated using the following equation: fertilization rate (%) = total number of two-cell embryos / total number of collected morphologically normal oocytes × 100. (C) The average number of fertilized oocytes obtained from each female mouse with a plug. (D) The efficiency was the average number of obtained fertilized oocytes from each female mouse used for copulation. Values are presented as the mean ± SD (n = 15–17). *p < 0.05 compared with pre ovulation. (PDF) [file pone.0281330.s002.pdf]
