# Supplementary material for: Synchronization of the ovulation and copulation timings increased the number of in vivo fertilized oocytes in superovulated female mice
Source: PLoS One. 2023 Feb 6;18(2):e0281330. doi: 10.1371/journal.pone.0281330 (PMC9901804; doi:10.1371/journal.pone.0281330)
Supplement: S1 Table — (DOCX) [file pone.0281330.s003.docx]

**S1 Table**

| **Reagent** | **mg/100 mL** | | |
| --- | --- | --- | --- |
|  | **cTYH** | **mHTF** | **KSOM** |
| **NaCl** | 697.6 | 593.8 | 555.0 |
| **KCl** | 35.6 | 35.0 | 18.5 |
| **MgSO_4_/7H_2_O** | 29.3 | 4.9 | 4.95 |
| **KH_2_PO_4_** | 16.2 | 5.4 | 4.75 |
| **NaHCO_3_** | 210.6 | 210.0 | 210.0 |
| **Sodium pyruvate** | 5.5 | 3.7 | 2.2 |
| **Glucose (D+)** | 100.0 | 50.0 | 3.6 |
| **CaCl_2_** | - | 57.0 | - |
| **CaCl_2_/2H_2_O** | 25.1 | - | 25.0 |
| **Sodium lactate (mL)** | - | 0.340 | 0.174 |
| **Penicillin G K salt** | - | 7.5 | 6.3 |
| **Streptomycin sulfate** | - | 5.0 | 5.0 |
| **10 mM EDTA (mL)** | - | - | 0.1 |
| **0.5% phenol red (mL)** | - | - | 0.1 |
| **L-Glutamine** | - | - | 14.6 |
| **MEM Essential Amino Acids solution (mL)** | - | - | 1.0 |
| **MEM Non-Essential Amino Acids solution (mL)** | - | - | 0.5 |
| **BSA** | - | 400 | 100 |
| **Methyl-β-cyclodextrin** | 98.3 |  |  |
| **Polyvinyl alcohol** | 100 |  |  |
